# Incidence of cancer in children residing in ten jurisdictions of the Mexican Republic: importance of the Cancer registry (a population-based study)

**DOI:** 10.1186/1471-2407-7-68

**Published:** 2007-04-19

**Authors:** Arturo Fajardo-Gutiérrez, Servando Juárez-Ocaña, Guadalupe González-Miranda, Virginia Palma-Padilla, Rogelio Carreón-Cruz, Manuel Carlos Ortega-Alvárez, Juan Manuel Mejía-Arangure

**Affiliations:** 1 Registro de Cáncer en Niños, Unidad de Investigación Médica en Epidemiología Clínica, Hospital de Pediatría, Centro Médico Nacional Siglo XXI, Ciudad de México

## Abstract

**Background:**

In 1996, Mexico started to register cases of childhood cancer. Here, we describe the incidence of cancer in children, residing in ten Mexican jurisdictions, who were treated by the Instituto Mexicano del Seguro Social (IMSS).

**Methods:**

New cases of childhood cancer, which were registered prospectively in nine principal Medical Centers of IMSS during the periods 1998–2000 (five jurisdictions) and 1996–2002 (five jurisdictions), were analyzed. Personnel were specifically trained to register, capture, and encode information. For each of these jurisdictions, the frequency, average annual age-standardized incidence (AAS) and average annual incidence per period by sex and, age, were calculated (rates per 1,000,000 children/years).

**Results:**

In total 2,615 new cases of cancer were registered, with the male/female ratio generally >1, but in some tumors there were more cases in females (retinoblastoma, germ cells tumors). The principal groups of neoplasms in seven jurisdictions were leukemias, central nervous system tumors (CNS tumors), and lymphomas, and the combined frequency for these three groups was 62.6 to 77.2%. Most frequently found (five jurisdictions) was the North American-European pattern (leukemias-CNS tumors-lymphomas). Eight jurisdictions had AAS within the range reported in the world literature. The highest incidence was found for children underless than five year of age. In eight jurisdictions, leukemia had high incidence (>50). The AAS of lymphomas was between 1.9 to 28.6. Chiapas and Guerrero had the highest AAS of CNS tumors (31.9 and 30.3, respectively). The frequency and incidence of neuroblastoma was low. Chiapas had the highest incidence of retinoblastoma (21.8). Germ-cell tumors had high incidence.

**Conclusion:**

The North American-European pattern of cancers was the principal one found; the overall incidence was within the range reported worldwide. In general but particularly in two jurisdictions (Yucatán and Chiapas), it will be necessary to carry out studies concerning the causes of cancer in children. Due to the little that is known about the incidence of cancer in Mexican children, it will be necessary to develop a national program to establish a cancer registry for the whole of the country.

## Background

Cancer in children differs from that in adults. In general, the principal groups of cancer in children are leukemias, lymphomas, and sarcomas, whereas in adults, the chief cancers are carcinomas [1]. For this reason, the manner of grouping the cancers for study is different. For children, the International Classification of Childhood Cancer (ICCC) is used; it is based on the morphology of the tumors and is composed of 12 main groups [2].

Childhood cancer encompasses, in general, the cases of cancer found in the cohort of 0–14 year-old children; however, some registers also include those found in the 15–19 year-old cohort [3]. At present, there is a controversy regarding the interpretation of trends because researchers in the United States of America (USA) and France [4,5] have shown no increase, whereas other researchers in Europe have shown an increase, in general, for acute lymphoblastic leukemia, Hodgkin's disease, central nervous system tumors (CNS tumors), and germ cell tumors [6-9]. Likewise, other investigators in the USA have demonstrated that there is a trend toward increase in the 0–9 year-old group [10].

Worldwide, the incidence of childhood cancer is generally between 100–180 per 1,000,000 children/year [11]. However, the incidence of the principal groups of cancers differs according to the country in which the study took place: For example, in the USA, the incidence of leukemia is 47.8 per 1,000,000 children/year [4], whereas in some countries, it can reach as high as 57.9 per 1,000,000 children/year [11]. Therefore, based on the three principal groups of cancer, various patterns of presentation of the disease have been described: 1) North American-European pattern (1^st^, leukemias; 2^nd^, CNS tumors; and 3^rd^, lymphomas); 2) Latin American pattern (1^st^, leukemias; 2^nd^, lymphomas; and 3^rd^, CNS tumors); and 3) African pattern (1^st^, lymphomas; 2^nd^, leukemias; and 3^rd^, CNS tumors or soft tissue sarcomas) [12].

By sex, the male/female ratio (M/F) is generally 1.2, but this value may vary according to country and to the type of cancer. For example, in many countries, the M/F for leukemias is 1.3; for Hodgkin lymphomas (HL), 2.5 or higher; and for renal tumors (Wilms tumor), <1. By age, the highest incidence of neoplasms in general is found in children less than five years old [12].

Other aspects of great relevance for the descriptive epidemiology of childhood cancer may be noted, such as the incidence in relation to environment (urban/rural), race, occupation of the parents, socio-economic stratum [12,13]. However, what should be most evident is that all these data are obtained, in general, from population-based cancer registries [3,5-9,11].

Due to the lack of a national program for the registry of childhood cancer, in Mexico data exist only for the incidence of cancer in those children who reside in Mexico City and who are entitled to treatment by the Instituto Mexicano del Seguro Social (IMSS). These data are obtained by means of hospital surveys. In other jurisdictions in the country, only the frequency of those cases treated in some hospitals is known, but the incidence is not known due to the absence of a specific registry for cancer and to the difficulties in obtaining the population at risk (denominators) [14-16]. This situation results in a grave problem, in that the magnitude of the problem of childhood cancer in the greater part of the jurisdictions of the country is unknown.

The aim of the present study is to present the incidence rates and the main groups of cancer in those children in ten jurisdictions of the Mexican Republic, who were treated by IMSS. IMSS is the principal institution that provides medical attention in Mexico, serving 50% of the population of the country. Socio-economic distinctions exist between the jurisdictions, which are reflected in their populations.

## Methods

### Type of study

Population-based, prospective study.

### Sources of information

IMSS provides medical attention to workers and their families. When a child of a worker develops some form of cancer, the child is sent to one of the specialized hospitals in the IMSS system (Medical Center-IMSS), which are distributed strategically throughout the country. The Medical Centers-IMSS are hospitals that have both the specialized personnel (oncologists, surgeons, hematologists, and pediatric pathologists) and the technology necessary to perform precise diagnosis and to give the treatment required for a child with cancer. IMSS keeps a register of the population entitled to receive medical attention at its facilities; therefore, the base population is known and it is feasible to obtain the incidence rate for the population under 15 years of age (Table 1). Not only do children with cancer receive medical treatment at no cost, but also when such children reside in areas in which no Medical Center-IMSS is located, they are referred to a Medical Center-IMSS closest to their place of residence, with IMSS covering the cost of transportation of these children for treatment.

**Figure 1:**
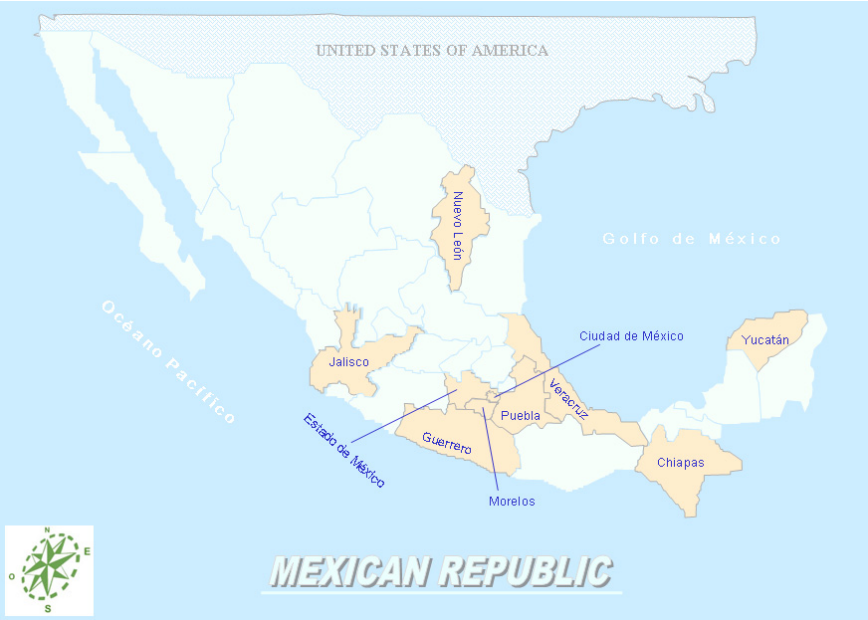
Jurisdictions of the Mexican Republic included in the study.

The information for this study was taken from two different sources of data. The first was the Registry of Childhood Cancer that was established in the Unit of Medical Research in Clinical Epidemiology of the Pediatric Hospital of the National Medical Center "Siglo XXI" of IMSS. This population-based registry of cases of cancer was started in 1996 and is ongoing [17]. Cases are registered for those children who are treated in Mexico City in either of the two hospitals, the Pediatric Hospital of the National Medical Center "Siglo XXI" and the General Hospital of the National Medical Center "La Raza", that IMSS maintains for providing free medical attention to those who, by right of being residents of Mexico City, are entitled to its services. These hospitals also treat children with cancer who are referred from other states of the Mexican Republic. The data were collected prospectively. Thus, the data used in this study represent the new cases of children with cancer, during the period 1996–2002, from Mexico City and from four states (Mexico, Morelos, Guerrero, and Chiapas), the first two located in the center of the country and last two, in the south (see Figure 1).

The second source of data was a hospital survey carried out during the period 1998–2000 in the remaining five Medical Centers-IMSS: one in the north of Mexico (Nuevo León); one in the west (Jalisco); one in the central part of the country (Puebla); one in the east (Veracruz); and one in the southeast (Yucatán) (see Figure 1). It should be noted that, in general, the populations in these jurisdictions are socio-economically distinct: Those in the North, West, Center, and East of the country are richer, whereas those in the South are poorer.

Because it is indispensable for providing treatment of any kind, the diagnosis of cancer for each patient who was included in the study, from both sources of data, was confirmed either by aspiration of bone marrow in the cases of leukemia or by histopathological study of the tumors for the remaining cases of different cancers. Only one patient, suspected of having hepatoblastoma, received chemotherapy without having had the diagnosis confirmed by biopsy. Unfortunately, the child died and a postmortem study was not done; however, the case was included in the analysis. For this reason, we consider that the histopathological validation of the registered cases was practically 100%.

### Study population

#### Numerators

The cases included in the numerators were those of patients who were treated at the different Medical Center-IMSS and who were residents in the above-mentioned jurisdictions. Only new cancer cases for children less than 15 years of age were included; prevalent cancer cases were excluded.

#### Denominators

IMSS holds a very precise record of the population entitled to receive medical care in its facilities; therefore, the population under 15 years of age and residing in the different jurisdictions during the period studied, the value needed to calculate the incidence, was obtained from the Coordination of Medical Treatment of the Medical Financing Board of IMSS. This is the only governmental agency that calculates all the populations with access to medical attention provided by the IMSS [18]. This information is updated every year by the IMSS. The recorded population of each of the jurisdictions during the study periods is shown in Table 1.

### Hospital units from which data were obtained

A child with cancer might be treated in different hospital services; therefore, the data were obtained from various pediatric services (Oncology, Hematology, Surgery, Neurosurgery, Endocrinology) of the different Medical Centers-IMSS selected, all of which have the infrastructure (well-trained personnel and technology) necessary to establish the diagnosis of malignant neoplasia precisely.

### Variables of the study

Prior to carrying out the study, we designed a form listing the variables of interest: those of the individual (*e.g*., sex, age at diagnosis, type of neoplasm), of place (residence according to the districts in which the Medical Centers-IMSS are located in the ten jurisdictions included in this study), and of time (age at diagnosis). All the foregoing variables were obtained by interviewing the parents or by reviewing the respective clinical records; such information was obtained for 100% of the cases included in this study.

### Procedure for obtaining, encoding, and validating the information

Prior to the actual gathering of data, nurses whose job it would be to collect and code such data, were specifically trained not only in obtaining the appropriate information from the hospital, but also in registering, coding, and verifying the type of cancer, site, and diagnosis of the cancer for each patient. In a pilot test at the last stage of the training, these nurses showed a good consistency in their coding of the data (unweighted Kappa = 0.85). However, the final encoding of the cases from both sources of data was carried out by personnel (three physicians and two nurses) of the Registry of Childhood Cancer of the Unit of Medical Research in Clinical Epidemiology of the Pediatric Hospital of the National Medical Center "Siglo XXI" of IMSS, all of whom had also been trained in the registry and coding of cases of cancer and who had shown an adequate consistency in coding the morphology, topography, and staging of the different cancers (unweighted Kappa = 0.90).

Therefore, for each of the nine Medical Centers-IMSS selected, a nurse was assigned to register all the new cases of malignant neoplasms in children. These nurses went every third day to the above-mentioned hospital services, with the aim of capturing all possible new cases of cancer. The nurse registered this information in a special file and then used the questionnaire to obtain pertinent information from the parents, which was needed for the study. Once a diagnosis of cancer had been confirmed, the nurse captured the data for inclusion in this study; those diagnoses not confirmed were discarded. If for any reason the patient had been released from the hospital, the nurse reviewed the patient's medical record in the archives of the respective hospital to learn the final diagnosis.

To encode the different cases of cancer, topographic and morphological coding was used; for the cases collected between 1996 through 1999, the second edition of the "International Classification of Diseases for Oncology" (ICD-O-2) was used, and for the cases between 2000 through 2002, the third edition (ICD-O-3) [19,20].

The collected information was then captured on two occasions with the aim of detecting any problems in capturing the data and, prior to analysis of the data, the respective databases were reviewed in search of duplicated cases and missing data.

Also, validation of the principal variables of the study (morphology and topography of the different types of cancer; age; sex) was performed by using the program, Child-Check, that was developed by the International Agency for Research on Cancer (IARC) [21], which evaluates the internal consistency of the individual cancer registries. The different types of cancer were grouped according to the ICCC [2].

### Case completeness

With the aim of having an objective evaluation of the case completeness in the different Jurisdictions included in the study, the expected incidence was calculated. To this end, we used the method that the North American Association of Central Cancer Registries follows to evaluate the case completeness when evaluating a central registry [22]. In this method, the ratio between the rate of the incidence of cancer and the mortality rate of cancer is calculated; therefore, the values not only of the incidence, but also of the mortality, must be as precise as is possible. The incidence (126.4 per million children/year) that we used was that of the jurisdiction of Mexico City and the mortality (71.7 per million children/year) was that reported by IMSS for children (0 to 14 years) during the period 1995–2000 [23]. With these data, we obtained the ratio for these rates (1.76) and we multiplied this value by the mortality for the period 1995–2000 that had been reported for each jurisdiction studied [23]. By using this method, we found that, in nine jurisdictions, the ratio of the observed/expected rates was equal to, or greater than 0.90; only in Jalisco, was it 0.71 (data not shown).

### Analysis

The absolute and the relative frequencies were obtained according to the type of neoplasm, to sex, and to age, with age being stratified into four subgroups (less then one year of age, one to four year-olds, five to nine year-olds, and ten to 14 year-olds). For each of the periods under study (1996–2002 and 1998–2000), the average annual age-standardized incidence (AAS) per period, both general and that for the different neoplasms grouped according to ICCC, were calculated by using the direct method and by taking the standard world population under 15 years of age as reference population [24]. Also calculated was the incidence according to sex, age, and place of residence for the selected jurisdictions of the Mexican Republic; the rates are given per 1,000,000 children/year. The protocol was approved by the Research and Ethics Coordination of the Pediatric Hospital, National Medical Center Century XXI, Instituto Mexicano del Seguro Social.

## Results

A total of 2,615 new cases of cancer in children was registered, of which 1,957 (74.8%) corresponded to the period 1996–2002 in the Pediatric Hospital of Medical Center "Siglo XXI" and Medical Center of "La Raza" (both in Mexico City) and 658 (25.2%) for the period 1998–2000 in the remaining Medical Centers. Table 2 shows the different types of neoplasms that were registered in each of the ten jurisdictions. Of the total number of cases in the study, 1,572 (60.1%) came from either Mexico City or the State of Mexico.

In general, the three types of neoplasms most commonly found were leukemias, with frequencies between 31.6% (Nuevo León) and 51.4% (Mexico State); CNS tumors, with frequencies between 8.8% (Puebla) and 18.6% (Nuevo León); and lymphomas, with frequencies between 2.0% (Yucatán) and 16.8%, (Guerrero). Only in Jalisco did CNS tumors and bone tumors have the same frequency (10.5%); in Yucatán, CNS tumors and germ cell tumors had the same frequency (9.8%) (Table 2).

As to specific type of neoplasms, among leukemias, acute lymphoblastic predominated; among lymphomas, non-Hodgkin (non-HL) predominated in six jurisdictions, and HL, in five; among CNS tumors, astrocytomas predominated in nine jurisdictions; between tumors of the sympathetic nervous system, neuroblastoma; between renal tumors, nephroblastoma; between hepatic tumors, hepatoblastoma; among bone tumors, osteosarcoma; among soft tissue sarcomas, rabdomyosarcoma predominated in six jurisdictions; and among germ cell tumors, malignant gonadal tumors. Due to the low frequency of carcinomas, it was difficult to establish with precision which type had the highest frequency (Table 2).

The AAS was found to range between 83.6 (Jalisco) and 203.5 (Chiapas). Notably, leukemias had the highest incidence in Chiapas (74.2); HL, in Puebla (11.9); non-HL, in Guerrero (19.9); CNS tumors, in Chiapas (31.9); neuroblastoma, in Guerrero (13.5); retinoblastoma, in Chiapas (21.8); renal tumors, in Morelos (10.8); hepatic tumors, in Puebla (11.3); bone tumors (12.2), soft tissue sarcomas (14.0), and carcinomas (5.9), all highest in Chiapas; and germ cell tumors, in Yucatán (10.6) (Table 3).

In general, the M/F ratio was higher than 1, with it being highest (1.4) in Jalisco; only in Yucatán was it less than 1. However, a greater variation in the M/F ratio was found within the different groups of neoplasms (Tables 4, 5, 6).

By age, the incidence for those <1 year of age varied between 38.6 and 330.7, with the highest found in the states of Chiapas, Puebla, and Morelos (330.7, 300.7, and 235.1, respectively); for 1–4 year-olds, the incidence varied between 106.7 and 263.0. The incidence was lower for 5–9 year-olds, ranging between 63.8 and 143.2, but rose again for 10–14 year-olds, varying between 82.1 and 173.2 (Tables 4, 5, 6).

In regard to the principal pattern of presentation, the North American-European pattern (1^st^, leukemias; 2^nd^, CNS tumors; and 3^rd^, lymphomas) was found in five jurisdictions; the Latin American pattern (1^st^, leukemias; 2^nd^, lymphomas; and 3^rd^, CNS tumors) in two jurisdictions; and in the other three jurisdictions, although leukemias and CNS tumors were predominant, bone tumors, germ cell tumors, or retinoblastoma were in second or third place (Jalisco, Yucatán, and Chiapas, respectively) (Tables 4, 5, 6).

## Discussion

A better understanding both of the descriptive epidemiology and of the underlying causes of childhood cancer would greatly aid the medical community and health authorities in Mexico to improve treatment for one of the most vulnerable populations, that of infants and children. Registering cases of cancer in children can be an enormous aid in understanding the epidemiology of these diseases. But it must be kept in mind that, to improve the precision of the estimates, it is necessary not only to have a completeness of coverage of cases, but also to have a quality control in an appropriate form in which to collect and register the data [22].

The quality should be evaluated in relation not only to the manner in which the data were collected but also, when a register is based on information obtained in the hospitals treating the children, to the rigorousness of the search for these cases in the different hospital services. Because cases so detected comprise the numerator for the estimation of the incidence rates, it has been recommended that medical records be reviewed in all those departments or services (*e.g*., Oncology, Hematology, Surgery, Endocrinology, Neurosurgery, Pathology, and Radiotherapy), in which medical attention may have been provided to a child with cancer and in which, therefore, a case of childhood cancer may have been registered [22]. Thus, for this study, the participating nurses searched exhaustively in these various hospital services for possible cases and performed a detailed registry of such information.

Another fundamental aspect is the reference population (or, the population at risk) from which the cases were taken. Because IMSS keeps a rigorous registry both of those entitled to treatment and of those treated in its facilities, the precise numbers of the populations that comprise the denominators needed to estimate the rates of incidence were obtained (Table 1) [18].

Because a population-based cancer registry is the "gold standard" for ascertaining the number of cancer cases that develops within a population, it is very difficult to evaluate if this ascertainment is complete. Children with cancer must be treated in specialized medical units and, at IMSS, they are treated in the Medical Centers-IMSS, because such centers have the necessary infrastructure and the medical personnel dedicated to the specialized treatment that children with cancer need. For this reason, we decided to select these centers to carry out this study. Additionally, the personnel that participated in the collection of data performed a strict inquiry into the number of new cases presented in the selected hospitals. We think that, through these actions, over 90% of the new cases that had developed in the selected jurisdictions were, in fact, registered. To support this, it was necessary that we had a proxy with which to assess the validity of the information registered. An indirect form of so doing was to compare the incidence rates obtained with those reported worldwide. For eight of the jurisdictions studied, the exceptions being Chiapas and Jalisco, the incidence was within the range of variability (100 to 180 per 1,000,000 children/year) reported in the world literature [11].

The incidence in Chiapas was higher than that reported worldwide. In theory, the contrary would have been expected, because it is more common to underestimate data for the numerator due to under-diagnosis and/or under-registration (or under-reporting) of cases. Thus, an incidence <100 per 1,000,000 children/year, as was found for Jalisco (82.6 per 1,000,000 children/year), would have been expected. Jalisco is an state in which, despite our best efforts to collect all the cases, an underestimation might be suspected. This situation can be remedied by carrying out further studies *ex profeso *or through the establishment of a Registry of Childhood Cancer in those states where no such Registry presently exists. Thus, at first glance, the high value for Chiapas may be thought to have resulted from the denominator having been underestimated. However, two factors led us to consider otherwise: Prior studies have demonstrated a high frequency of cancers, such as retinoblastoma, in Chiapas [16,25,26], and the pathologists of the Pediatric Hospital of Medical Center "Siglo XXI" have commented on their impression that cases of the rarest types of cancer in children were found in Chiapas (personal communication). Such was the case in the present work as, in Chiapas, both the frequency (3.5%) and the incidence (5.9 per 1,000,000 children/year) of carcinomas (usually the least frequent among cases of childhood cancers) were found not only to be higher than those of any of the other ten jurisdictions, but also to be one of the highest reported in the world [5,6,11].

We corroborated the foregoing by calculating the ratio of the observed rate to the expected rate for each Jurisdiction studied [22]. As was mentioned in Methods, we found that, in nine jurisdictions, the ratio of the observed and the expected rates was equal to, or greater than, 0.90; only in Jalisco, was it 0.71. With these data, we corroborated that we had an underestimation of the registered cases in Jalisco and that the incidence in Chiapas is one of the highest in the world [11]. The finding that one state had such a distinct profile of cases of childhood cancer underscores the necessity of developing analytical studies with the aim of understanding the risk factors that this pediatric population has in developing cancer.

Other indications of the validity of the data presented here were that both the frequency and the incidence of the principal types of cancer, the profile of the age at presentation (greatest incidence in those <5 years of age, falling off at 5–9 years, and rising again at 10–14 years), and the M/F ratio were consistent with the values reported for other countries [3,5-8,11]. From the foregoing, we consider that, based on the hospitals selected for the study and on the manner in which the data were collected and the results obtained, our data on the frequency and incidence of childhood cancer in the jurisdictions in this study (with the possible exception of Jalisco) have the greatest precision when compared to data previously reported [14-16]. However, we must continue to perform a quality registry of the cases of cancer in the jurisdictions studied, because when a great number of cases, especially well-registered cases, are used for the calculations, the precision of the resulting incidence in the studied population will be increased.

Although IMSS provides medical attention to 50% of the Mexican population (thus, to approximately 50% of the population of Mexican children), only workers and their family members are covered. Because the remainder of the population, 10% of which are considered to live in extreme poverty, are attended by other institutions (Ministry of Health, the Armed Forces, Instituto de Seguridad Social al Servicio de los Trabajadores del Estado), it will be necessary to perform studies specifically with these populations in order to know the incidence of cancer in Mexican children.

It is interesting that no unique pattern for the principal groups of cancer (leukemias, CNS tumors, and lymphomas) was found; in the present study, the North American-European pattern was found in six of the ten jurisdictions studied, with the data from Nuevo Leon most notably demonstrating this pattern. Data from Morelos and Puebla clearly showed the Latin-American pattern. Jalisco, Chiapas, and Yucatán have another pattern. From these data, the question arises as to whether the different patterns in the presentation of different cancer groups were due to the use of a detailed cancer registry, or to distinct or changing risk factors, or to a combination of the two. It should be noted that, in the present study, the collection of data was prolective; there is no doubt that the collection was more detailed and more precise compared to the data in previous studies in which the collection was retrospective [14-16].

However, we may also consider the possibility that there may be distinct risk factors encountered in the jurisdictions studied, because the jurisdiction located in the north (Nuevo León); the west (Jalisco); the center (Mexico City, State of Mexico, Morelos, Puebla); or the south or southeast of Mexico (Guerrero, Veracruz, Chiapas, Yucatán) generally have different patterns of disease and different socio-economic conditions. Those jurisdictions in the North have a pathology similar to that of North American children; for those in the Western, Center, and most especially, in the South, the principal pathologies are the infectious type and nutritional deficiencies. Similarly, the states in the north and in the center of the country have better socio-economic conditions compared to those of the states in the South, the poorest being Guerrero, Chiapas [27,28]. This leads to the supposition that distinct risk factors may act on the presentation of the different cancers. It would, therefore, be interesting to carry out analytical studies to establish what the possible risk factors are and to determine if the disease patterns are due to the improved registry of cases, to these distinct risk factors, or to both.

In regard to the predominant neoplasms, leukemias, HD, neuroblastoma, retinoblastoma, and germ cell tumors deserve special comment. For leukemias, in general, the children in this study had some of the highest frequencies of leukemias in the world (seven jurisdictions having a frequency >40%) and, consequently, also had some of the highest incidence (eight jurisdictions with an incidence >50 per 1,000,000 children/year), with values comparable to those of children in Canada, of Hispanic and Black children in Los Angeles, and of children in Costa Rica and Ecuador. For other continents, the data are similar for the children of Denmark, Australia, Hong Kong, and Singapore [11]. These data underscore the necessity of developing analytical studies with the objective of determining and studying possible risk factors. In particular, due to how widespread it is in the Mexican population, tobacco smoking would be an appropriate choice to study in relation to the prevalence of leukemia. In a study on children with Down's Syndrome who reside in Mexico City, it has been shown that if the father smokes while the mother is pregnant, the child has a higher risk of developing leukemia [29].

For HL, the lowest incidence was found in Yucatán, and Jalisco (1.9 and 2.5 per 1,000,000 children/year, respectively); in the remaining states, the incidence ranged between 3.6 per 1,000,000 children/year (Mexico City) and 11.9 per 1,000,000 children/year (Puebla), values similar to those for children in North America and Europe [11]. It is noteworthy that, in comparison to previous reports for the children residing in Mexico City, not only the frequency (10.7, 9.0, and 3.1% in 1991, 1992–1993, and 1996–2002, respectively) but also the incidence (6.5, 7.7, and 3.6 per 1,000,000 children/year for these same time periods) apparently fell spontaneously [14,15,26]. This phenomenon is consistent with that mentioned by Linet *et al*. (1999) for children in the United States [4]. Because the Epstein-Barr virus has been mentioned as a possible causal agent [30] in the etiology of the HL, it will be interesting to perform an ecological study to determine if any correlation exists between the use of antivirals, which at present are used indiscriminately throughout much of Mexico, and the incidence of HL. Data from such a study would either prove or disprove the hypothesis that the apparently spontaneous fall in HL in the children of Mexico City may be due to the excessive use of antivirals [26].

It is interesting that, in developed countries, the frequency of neuroblastoma is 5 to 10% and the incidence is 8.0 to 15.0 per 1,000,000 children/year, values that contrast with those in developing countries (frequency, 3 to 5%; incidence, is 3.5 to 9.3 per 1,000,000 children/year) [11]. The data obtained in our study are similar to those from the developing countries, with eight jurisdictions having a frequency <5% and seven jurisdictions having an incidence less than 5 per 1,000,000 children/year. In countries such as Japan, where screening tests are carried out in an attempt to achieve an early diagnosis, it has been shown that, of the cases detected by this method, 59% had spontaneous regression [31]. It is likely that, in Mexico and in countries having similar socio-economic development, there really is a lower frequency of cases of neuroblastoma, or whether there are cases that are not diagnosed but that spontaneously regress [32].

Between 20 to 40% of retinoblastoma, an embryonic tumor, may have a hereditary component [33]. It has been shown that not only the frequency but also the incidence of retinoblastoma is higher in developing countries than in developed countries [3-8,11]. These data are reflected in the present study, in that the incidence in the jurisdictions in the south of Mexico, areas than are poorer than those in the north of the country [28], had higher incidence. Corroborating the results of previous studies carried out by our group, the present data showed that Chiapas in the south of Mexico had one of the highest reported frequencies of retinoblastoma [16,25,26] and, in addition, had the highest incidence (21.8 per 1,000,000 children/year). Also, the data are consistent with a recent study carried out in a population in the south of Mexico, where reduction of the mother's intake of vegetables and fruits during the pregnancy increased the risk of developed sporadic retinoblastoma in their children [34].

In our study, germ cell tumors had a frequency and incidence that were slightly greater than the values in the international literature, in which both the frequency (3.0 to 6.8%) and the incidence (2.5 to 9.6 per 1,000,000 children/year) are generally higher in Asian countries [11]. With the exception of Guerrero, the frequencies in the jurisdictions ranged between 3.7 and 9.8% and the incidence between 2.3 and 10.6 per 1,000,000 children/year. Worldwide, tumors of the gonads are the most frequently found (40 to 50%) of the germ cells tumors, whereas in our data, although they were the most frequent, the proportion was higher (43.2 to 100%). However, because the highest frequency and incidence was found in Yucatán, the jurisdiction with the fewest cases in the study, this frequency may be random. Therefore, it will be necessary to perform studies with a greater number of cases in order to confirm these findings.

A Registry of Childhood Cancer is extremely important means of assessing both the frequency and the incidence of neoplasms in the pediatric population, values that are not known in any great detail in Mexico at present. Knowledge of the frequency and the incidence of childhood cancer have other very valuable uses:

1) In the clinical setting, knowing the frequency of diseases serves to establish the pretest probability that a patient has upon arrival at the hospital. Such probability increases or decreases depending on the symptoms presented, on the sensitivity and specificity of the test(s) requested, and on the result (positive or negative) of such test(s) [35]. From the present data, for a child who resides in Mexico City and for whom the parents sought medical attention or who was referred to the tertiary care pediatric hospital upon suspicion of cancer, the pretest probability (without taking into account the child's symptoms) for having a leukemia is 43.7%; a lymphoma, 12.0%; or a CNS tumor, 13.4% (Table 2). This shows the importance of learning the frequency of the different types of cancer (or, for that matter, of illnesses in general) that are present in Mexican children.

2) Knowledge of the frequency and incidence of childhood cancer can aid in the planning of future health services, in that it provides a basis not only for estimating the personnel (physicians, nurses, laboratory technicians, etc.) necessary to provide medical care to these children, but also for allocating the equipment, pharmaceuticals, and laboratory consumables necessary for the ideal treatment of children with cancer [36]. For example, when planning the medical attention needed to treat childhood cancer in Mexico City, it should be taken into account that the data suggest that the great majority (43.7%) of these children will have some type of leukemia and that, yearly, 55.4 cases per million children residing in this jurisdiction may be expected (Table 3). Similar predictions may be made for the children in the other jurisdictions studied.

From the foregoing, it is imperative that, in Mexico, a definitive registry of childhood cancer be established in all hospitals that treat children with cancer. This should be done *ex profeso *for cases of cancer in the pediatric population, because cancer in children is distinct from that in adults. The coding and grouping are quite different; hence, personnel who work on these registries must have the specialized training required to maintain the quality of the registry [37,38]. Such a Cancer Registry would be the first step towards a better understanding of the epidemiology of cancer in the pediatric population and, in the future, towards the prevention and control of cancer, as has been seen in countries that have established national programs for the registry of cancer [39].

We think that to be most effective, when establishing a Registry of Childhood Cancers, the registering of cases of pediatric cancer be made mandatory. In general, of the total pediatric population (supposing an average incidence of 130 per 1,000,000 children/year), 4,000 to 4,500 cases of childhood cancer are presented annually in Mexico. Therefore, IMSS each year will treat approximately between 2,000 and 2250 cases that could well be reported to, and included in, the Registry of Childhood Cancers of the hospitals of the Medical Centers-IMSS. Thus, these children and the society in general would reap the benefits obtained when cases of childhood cancer are properly registered.

## Conclusion

We conclude that the North American-European pattern of cancer was the principal one encountered and that the general incidence was within the range reported in the world literature. The incidence of leukemias was high in the majority of the jurisdictions studied, whereas the incidence of neuroblastoma was low. The highest incidence of retinoblastoma was found in Chiapas, one of the poorest jurisdictions. In general but particularly in two jurisdictions (Yucatán and Chiapas), it will be necessary to develop studies concerning the causes of cancer in children. We think that a detailed and mandatory Registry of Childhood Cancer must be established in all hospitals that provide medical attention to these children. We consider that this would be a great step in learning of incidence of these diseases in Mexico.

## Competing interests

The author(s) declare that they have no competing interests.

## Authors' contributions

AFG conceived and designed the study, analyzed the data, corrected the final manuscript, and provided guidance to all aspects of this project. SJO recoded, analyzed the data, and wrote the first draft of the manuscript. GGM, VPP, RCC and MCOA recoded, revised and analyzed the data base, and participated in the interpretation of results. JMMA analyzed the data, participated in the interpretation of results, critically revised the manuscript, and provided guidance in some aspects of the project. All authors read and approved the final manuscript.

## Pre-publication history

The pre-publication history for this paper can be accessed here:



## Figures and Tables

**Table 1:** Population at risk of children served by IMSS and assigned a family doctor in ten jurisdictions of the Mexican Republic during the study periods

		**Age group (years)**					**Sex**	
		
Jurisdictions	**Periods**	**<1**	**1 – 4**	**5 – 9**	**10 – 14**	**0 – 14**	**Male**	**Female**
Chiapas	1996–2002	36 291	186 336	272 335	248 197	743 159	375 498	367 661
México City	"	375 761	1 597 012	2 049 305	1 907 323	5 929 401	3 024 225	2 905 176
Guerrero	"	38 352	206 442	282 347	221 690	748 831	378 945	369 886
México State	"	463 520	1 962 182	2 538 935	2 298 797	7 263 434	3 693 756	3 569 678
Morelos	"	38 281	218 981	284 204	249 268	790 734	404 759	385 975
Jalisco	1998–2000	138 929	656 340	799 563	718 287	2 313 119	1 180 648	1 132 471
Nuevo León	"	129 612	522 439	592 491	490 141	1 734 683	885 697	848 986
Puebla	"	36 580	219 003	281 628	244 852	782 063	397 323	384 740
Veracruz	"	48 841	282 157	400 989	378 902	1 110 889	566 340	544 549
Yucatán	"	27 506	149 060	181 721	154 519	512 806	261 102	251 704

**Source**: Coordinación de Atención Médica. Estructura de Población Adscrita a Médico Familiar, 1996–2002. Instituto Mexicano del Seguro Social, México (Reference 18).

**Table 2:** Frequency of cancer in children served by the IMSS and residing in ten jurisdictions of the Mexican Republic during the periods 1996–2002 and 1998–2000

***Diagnostic Group***	**1996–2002**										**1998–2000**											
	**CHIS**		**MC**		**GRO**		**MS**		**MOR**		**JAL**		**NL**		**PUE**		**VER**		**YUC**		Overall	
	**n**	**%**	**n**	**%**	**n**	**%**	**n**	**%**	**n**	**%**	**n**	**%**	**n**	**%**	**n**	**%**	**n**	**%**	**n**	**%**	**n**	**%**
**I. Leukemias**	**54**	**37.8**	**321**	**43.7**	**43**	**34.4**	**431**	**51.4**	**54**	**46.2**	**97**	**50.8**	**56**	**31.6**	**56**	**49.1**	**60**	**48.0**	**25**	**49.0**	**1197**	**45.8**^1^
*+ Acute lymphoblastic*	*37*	*68.5*	*249*	*77.6*	*35*	*81.4*	*352*	*81.7*	*42*	*77.7*	*78*	*80.3*	*53*	*94.6*	*48*	*85.7*	*46*	*76.7*	*18*	*72.0*	*958*	*80.0*^2^
*+ Acute non-lymphoblastic*	*16*	*29.6*	*58*	*18.1*	*7*	*16.3*	*70*	*16.3*	*12*	*22.3*	*16*	*16.5*	*3*	*5.4*	*8*	*14.3*	*11*	*18.3*	*5*	*20.0*	*206*	*17.2*
																						
**II. Lymphomas**^3^	**15**	**10.5**	**88**	**12.0**	**21**	**16.8**	**75**	**9.0**	**15**	**12.9**	**17**	**8.9**	**22**	**12.4**	**19**	**16.7**	**16**	**12.8**	**1**	**2.0**	**289**	**11.1**
*+ Hodgkin lymphomas*	*6*	*40.0*	*23*	*25.9*	*7*	*33.3*	*36*	*47.8*	*3*	*20.2*	*6*	*34.8*	*10*	*45.2*	*10*	*52.7*	*9*	*56.3*	*1*	*100.0*	*111*	*38.4*
*+ Non Hodgkin lymphomas*	*7*	*46.7*	*53*	*60.4*	*9*	*42.9*	*35*	*46.7*	*9*	*59.7*	*7*	*41.6*	*8*	*36.3*	*8*	*41.9*	*4*	*25.0*	*0*	*0.0*	*140*	*48.4*
*+ Burkitt lymphoma*	*2*	*13.3*	*9*	*10.3*	*5*	*23.8*	*3*	*4.4*	*3*	*20.2*	*4*	*23.6*	*3*	*13.7*	*1*	*5.4*	*3*	*18.8*	*0*	*0.0*	*33*	*11.4*
																						
**III. CNS tumors**	**21**	**14.7**	**98**	**13.4**	**23**	**18.4**	**75**	**9.0**	**11**	**9.5**	**20**	**10.5**	**33**	**18.6**	**10**	**8.8**	**19**	**15.2**	**5**	**9.8**	**315**	**12.0**
*+ Ependymomas*	*3*	*14.3*	*14*	*14.2*	*3*	*13.0*	*10*	*13.3*	*1*	*9.5*	*1*	*4.9*	*2*	*5.9*	*0*	*0.0*	*4*	*21.1*	*0*	*0.0*	*38*	*12.1*
*+ Astrocytomas*	*9*	*42.9*	*52*	*53.0*	*13*	*56.5*	*31*	*41.1*	*5*	*45.3*	*5*	*24.8*	*14*	*42.5*	*4*	*39.8*	*5*	*26.3*	*1*	*20.4*	*139*	*44.1*
*+ IIET*	*5*	*23.8*	*25*	*25.4*	*6*	*26.1*	*25*	*33.3*	*3*	*27.4*	*4*	*20.3*	*12*	*36.6*	*1*	*10.2*	*5*	*26.3*	*2*	*39.8*	*88*	*27.9*
																						
**IV. Neuroblastoma and others**	**3**	**2.1**	**22**	**3.0**	**8**	**6.4**	**16**	**1.9**	**2**	**1.7**	**4**	**2.1**	**11**	**6.2**	**1**	**0.9**	**1**	**0.8**	**3**	**5.9**	**71**	**2.7**
*+ Neuroblastoma*	*3*	*100.0*	*21*	*96.7*	*8*	*100.0*	*14*	*89.5*	*2*	*100.0*	*4*	*100.0*	*10*	*90.3*	*1*	*100.0*	*1*	*100.0*	*3*	*100.0*	*66*	*93.0*
																						
**V. Retinoblastoma**	**12**	**8.4**	**26**	**3.5**	**5**	**4.0**	**33**	**3.9**	**6**	**5.1**	**3**	**1.6**	**7**	**4.0**	**4**	**3.5**	**6**	**4.8**	**3**	**5.9**	**105**	**4.0**
																						
**VI. Renal tumors**	**4**	**2.8**	**30**	**4.1**	**7**	**5.6**	**33**	**4.0**	**7**	**6.0**	**5**	**2.6**	**10**	**5.6**	**2**	**1.8**	**6**	**4.8**	**2**	**3.9**	**106**	**4.1**
*+ Nephroblastoma*	*4*	*100.0*	*28*	*97.5*	*7*	*100.0*	*33*	*100.0*	*7*	*100.0*	*5*	*100.0*	*10*	*100.0*	*2*	*100.0*	*6*	*100.0*	*2*	*100.0*	*105*	*99.1*
																						
**VII. Hepatic tumors**	**3**	**2.1**	**19**	**2.6**	**0**	**0.0**	**10**	**1.2**	**1**	**0.9**	**4**	**2.1**	**3**	**1.7**	**6**	**5.3**	**1**	**0.8**	**1**	**2.0**	**48**	**1.8**
*+ Hepatoblastoma*	*2*	*66.7*	*17*	*88.5*	*0*	*0.0*	*9*	*91.7*	*1*	*100.0*	*4*	*100.0*	*3*	*100.0*	*6*	*100.0*	*1*	*100.0*	*1*	*100.0*	*44*	*91.7*
																						
**VIII. Bone tumors**	**10**	**7.0**	**39**	**5.3**	**8**	**6.4**	**47**	**5.6**	**7**	**6.0**	**20**	**10.5**	**5**	**2.8**	**5**	**4.4**	**3**	**2.4**	**4**	**7.8**	**148**	**5.7**
*+ Osteosarcomas*	*8*	*80.0*	*27*	*70.0*	*5*	*62.5*	*36*	*76.8*	*4*	*56.7*	*19*	*94.9*	*4*	*80.0*	*3*	*59.1*	*2*	*66.7*	*2*	*50.0*	*110*	*74.3*
																						
**IX. Soft tissue sarcomas**	**9**	**6.3**	**41**	**5.6**	**6**	**4.8**	**44**	**5.3**	**7**	**6.0**	**13**	**6.8**	**14**	**7.9**	**5**	**4.4**	**4**	**3.2**	**2**	**3.9**	**145**	**5.5**
*+ Rhabdomyosarcomas*	*4*	*44.4*	*25*	*61.2*	*1*	*16.7*	*27*	*60.4*	*4*	*56.7*	*8*	*61.8*	*12*	*85.4*	*2*	*40.9*	*4*	*100.0*	*1*	*50.0*	*88*	*60.7*
*+ Fibrosarcomas*	*4*	*44.4*	*9*	*21.9*	*1*	*16.7*	*8*	*18.9*	*1*	*15.0*	*0*	*0.0*	*1*	*7.3*	*0*	*0.0*	*0*	*0.0*	*0*	*0.0*	*24*	*16.6*
																						
**X. Germ cell tumors**	**7**	**4.9**	**45**	**6.1**	**2**	**1.6**	**67**	**8.0**	**5**	**4.3**	**7**	**3.7**	**14**	**7.9**	**6**	**5.3**	**9**	**7.2**	**5**	**9.8**	**167**	**6.4**
*+ Malignant gonadal*	*4*	*57.1*	*35*	*77.8*	*2*	*100.0*	*49*	*72.5*	*2*	*39.5*	*3*	*43.2*	*10*	*70.9*	*4*	*66.0*	*4*	*44.4*	*4*	*79.6*	*117*	*70.1*
																						
**XI. Carcinomas**	**5**	**3.5**	**5**	**0.7**	**2**	**1.6**	**7**	**0.7**	**2**	**1.7**	**1**	**0.5**	**2**	**1.1**	**0**	**0.0**	**0**	**0.0**	**0**	**0.0**	**24**	**0.9**
																						
**TOTAL**	**143**	**100**	**734**	**100**	**125**	**100**	**838**	**100**	**117**	**100**	**191**	**100**	**177**	**100**	**114**	**100**	**125**	**100**	**51**	**100**	**2615**	**100**

^1^Intergroup frequency (%); ^2^Intragroup frequency (%), only the frequencies of principal subgroups are presented; ^3^63 cases of Histiocytosis were not included; IMSS, Instituto Mexicano del Seguro Social; n, Number of cases; CNS, Central Nervous System; IIET, Intracraneal and Intraespinal Embryonal Tumors; CHIS, Chiapas; MC, Mexico City; GRO, Guerrero; MS, Mexico State; MOR, Morelos; JAL, Jalisco; NL, Nuevo León; PUE, Puebla; VER, Veracurz; YUC, Yucatán.

**Table 3:** Average annual age-standardized incidence per period^1 ^of malignant neoplasms in children served by the IMSS and residing in ten jurisdictions of the Mexican Republic during the periods 1996–2002 and 1998–2000

***Diagnostic group***	**1996–2002**					**1998–2000**					**Overall**	
	**CHIS**	**MC**	**GRO**	**MS**	**MOR**	**JAL**	**NL**	**PUE**	**VER**	**YUC**	**Crude**	**ASR**
**I. Leukemias**	**74.2**	**55.4**	**58.8**	**60.2**	**66.8**	**42.5**	**32.5**	**72.9**	**54.8**	**50.1**	**54.6**	**55.4**
*+ Acute lymphoblastic*	*51.8*	*43.2*	*47.3*	*49.3*	*52.5*	*34.3*	*30.7*	*62.3*	*42.6*	*35.8*	*43.7*	*44.5*^2^
*+ Acute non-lymphoblastic*	*21.2*	*9.8*	*10.1*	*9.6*	*14.3*	*6.8*	*1.8*	*10.6*	*8.9*	*9.7*	9.4	*9.3*
												
**II. Lymphomas**^3^	**18.6**	**14.4**	**28.6**	**9.9**	**18.9**	**7.2**	**12.7**	**22.7**	**12.9**	**1.9**	**13.2**	**12.8**
*+ Hodgkin lymphomas*	*8.0*	*3.6*	*8.6*	*4.6*	*3.7*	*2.5*	*5.8*	*11.9*	*7.4*	*1.9*	*5.1*	*4.8*
*+ Non Hodgkin lymphomas*	*8.3*	*8.9*	*12.8*	*4.7*	*11.1*	*3.0*	*4.6*	*9.4*	*3.2*	*0.0*	*6.4*	*6.3*
*+ Burkitt lymphoma*	*2.4*	*1.4*	*7.1*	*0.4*	*3.9*	*1.7*	*1.7*	*1.4*	*2.4*	*0.0*	*1.5*	*1.5*
												
**III. CNS tumors**	**31.9**	**16.5**	**30.3**	**10.3**	**15.7**	**8.9**	**19.2**	**12.1**	**16.8**	**9.5**	**14.4**	**14.4**
*+ Ependymomas*	*5.4*	*2.4*	*4.3*	*1.4*	*1.2*	*0.5*	*1.2*	*0.0*	*3.5*	*0.0*	*1.7*	*1.8*
*+ Astrocytomas*	*12.5*	*8.8*	*16.2*	*4.1*	*7.4*	*2.3*	*8.2*	*5.2*	*4.6*	*1.8*	*6.3*	*6.3*
*+ IIET*	*7.8*	*4.2*	*8.3*	*3.5*	*3.9*	*2.0*	*7.1*	*1.2*	*4.6*	*3.8*	*4.0*	*4.1*
												
**IV. Neuroblastoma and others**	**5.5**	**4.1**	**13.5**	**2.4**	**4.0**	**1.9**	**6.5**	**1.2**	**0.8**	**6.2**	**3.2**	**3.6**
*+ Neuroblastoma*	5.5	3.9	13.5	2.1	4.0	1.9	5.9	1.2	0.8	6.2	3.2	3.4
												
**V. Retinoblastoma**	**21.8**	**5.1**	**8.0**	**5.2**	**9.7**	**1.4**	**4.1**	**7.1**	**6.6**	**5.9**	**4.8**	**5.5**
												
**VI. Renal tumors**	**5.2**	**5.4**	**10.1**	**5.0**	**10.8**	**2.4**	**5.7**	**3.3**	**6.6**	**4.1**	**4.8**	**5.2**
*+ Nephroblastoma*	*5.2*	*5.3*	*10.1*	*5.0*	*10.8*	*2.4*	*5.7*	*3.3*	*6.6*	*4.1*	4.8	*5.2*
												
**VII. Hepatic tumors**	**4.5**	**3.5**	**0.0**	**1.5**	**1.4**	**2.1**	**1.8**	**11.3**	**1.1**	**2.1**	**2.2**	**2.5**
*+ Hepatoblastoma*	*3.3*	*3.2*	*0.0*	*1.3*	*1.4*	*2.1*	*1.8*	*11.3*	*1.1*	*2.1*	*2.0*	*2.3*
												
**VIII. Bone tumors**	**12.2**	**6.1**	**9.8**	**6.1**	**8.3**	**8.1**	**3.0**	**5.9**	**2.6**	**7.5**	**6.7**	**6.3**
*+ Osteosarcomas*	*9.9*	*4.2*	*6.2*	*4.6*	*4.6*	*7.7*	*2.4*	*3.6*	*1.8*	*3.7*	*5.0*	*4.7*
												
**IX. Soft tissue sarcomas**	**14.0**	**7.1**	**9.3**	**6.1**	**9.7**	**5.7**	**8.2**	**6.1**	**4.0**	**3.8**	**6.6**	**6.8**
*+ Rhabdomyosarcomas*	*6.7*	*4.3*	*1.3*	*3.8*	*5.4*	*3.7*	*7.0*	*2.4*	*4.0*	*1.8*	4.0	*4.2*
*+ Fibrosarcomas*	*5.7*	*1.6*	*1.3*	*1.1*	*2.0*	*0.0*	*0.6*	*0.0*	*0.0*	*0.0*	1.1	*1.1*
												
**X. Germ cell tumors**	**9.7**	**7.7**	**2.3**	**9.6**	**6.5**	**3.0**	**8.2**	**8.3**	**8.0**	**10.6**	**7.6**	**7.9**
*+ Malignant gonadal*	*6.2*	*6.0*	*2.3*	*7.1*	*2.8*	*1.2*	*5.9*	*5.0*	*3.4*	*8.9*	*5.3*	*5.5*
												
**XI. Carcinomas**	**5.9**	**0.9**	**2.8**	**0.9**	**2.3**	**0.4**	**1.2**	**0.0**	**0.0**	**0.0**	**1.1**	**1.0**
												
**TOTAL**	**203.5**	**126.4**	**173.5**	**117.3**	**154.3**	**83.6**	**103.0**	**150.7**	**114.3**	**101.8**	**119.2**	**121.5**

^1^Rates per 1,000,000 child/years; ^2^Only the incidence of principal subgroups are presented; ^3^63 cases of Histiocytosis were not included; IMSS, Instituto Mexicano del Seguro Social, n, Number of cases; CNS, Central Nervous System; IIET, Intracraneal and Intraespinal Embryonal Tumors; STS, Soft Tissue Sarcomas; CHIS, Chiapas; MC, Mexico City; GRO, Guerrero; MS, Mexico State; MOR, Morelos; JAL, Jalisco; NL, Nuevo León; PUE, Puebla; VER, Veracurz; YUC, Yucatán.

**Table 4:** Average annual and age-standardized incidence per period^1 ^of five principal malignant neoplasms in children served by IMSS and residing in the jurisdictions of Nuevo León, and Jalisco

Diagnostic group	**Age group (years)**				**Sex**				
	**<1**	**1–4**	**5–9**	**10–14**	**M**	**F**	**AAI**	**AAS**	**M/F^2^**
**Nuevo León 98-00 (cases)**	**N = 5**	**n = 85**	**n = 40**	**n = 47**	**n = 96**	**n = 81**	**n = 177**	**n = 177**	**96/81**
**1. Leucemias**	7.7	57.4	25.3	20.4	32.7	31.8	32.3	32.5	1.1
**2. CNS tumors**	7.7	23.0	11.8	26.5	21.5	16.5	19.0	19.2	1.4
**3. Lymphomas**	7.7	13.4	10.1	16.3	13.5	11.8	12.7	12.7	1.2
*- Hodgkin lymphomas*	*0.0*	*5.7*	*3.4*	*10.2*	*4.5*	*7.1*	*5.8*	*5.8*	*0.7*
*- Non Hodgkin lymphomas*	*7.7*	*7.7*	*6.8*	*6.1*	*9.0*	*4.7*	*6.9*	*6.9*	*2.0*
**4. Soft-tissue sarcomas**	0.0	19.1	3.4	4.1	10.2	5.9	8.1	8.2	1.8
**5. Germ cell tumors**	0.0	11.5	3.4	12.2	5.6	10.6	8.1	8.2	0.6
**Total**	**38.6**	**162.7**	**67.5**	**95.9**	**108.4**	**95.4**	**102.0**	**103.0**	**1.2**

									
**Jalisco 98-00 (cases)**	**N = 11**	**n = 70**	**N = 51**	**n = 59**	**n = 110**	**n = 81**	**n = 191**	**n = 191**	**110/81**
**1. Leucemias**	14.4	67.0	31.3	36.2	52.5	30.9	41.9	42.5	1.8
**2. CNS tumors**	21.6	7.6	7.5	8.4	10.2	7.1	8.6	8.9	1.5
**3. Bone Tumors**	0.0	0.0	12.5	13.9	4.2	13.2	8.6	8.1	0.3
**4. Lymphomas**	0.0	7.6	5.0	11.1	8.5	6.2	7.3	7.2	1.4
*- Hodgkin lymphomas*	*0.0*	*1.5*	*2.5*	*4.2*	*3.4*	*1.8*	*2.6*	*2.5*	*2.0*
*- Non Hodgkin lymphomas*	*0.0*	*6.1*	*2.5*	*7.0*	*5.1*	*4.4*	*4.8*	*4.7*	*1.2*
**5. Soft-tissue sarcomas**	7.2	7.6	2.5	7.0	6.8	4.4	5.6	5.7	1.6
**Total**	**79.2**	**106.7**	**63.8**	**82.1**	**93.2**	**71.5**	**82.6**	**86.3**	**1.4**

^1^Rates per 1,000,000 child/years; ^2^M/F = Ratio male/female; AAI = Average annual incidence; AAS = Average annual age-standardized incidence; CNS = Central Nervous System.

**Table 5:** Average annual and age-standardized incidence per period^1 ^of five principal malignant neoplasms in children served by IMSS and residing in the jurisdictions of Mexico City, State of Mexico, Morelos, and Puebla

	**Age group (years)**				**Sex**				
**Diagnostic group**	**<1**	**1–4**	**5–9**	**10–14**	**M**	**F**	**AAI**	**AAS**	**M/F**^2^
**México City 92-02 (cases)**	**n = 44**	**n = 266**	**n = 220**	**n = 204**	**n = 392**	**n = 342**	**n = 734**	**n = 734**	**392/342**
**1. Leukemias**	37.3	78.9	50.7	40.4	58.2	49.9	54.1	55.4	1.2
**2. CNS tumors**	10.6	18.2	15.6	17.3	16.2	16.9	16.5	16.5	1.0
**3. Lymphomas**	0.0	13.1	16.1	17.8	20.8	8.6	14.8	14.4	2.5
*- Hodgkin lymphomas*	*0.0*	*1.3*	*2.9*	*7.9*	*5.9*	*1.7*	*3.9*	*3.6*	*3.6*
*- Non Hodgkin lymphomas*	*0.0*	*11.9*	*13.2*	*10.0*	*14.9*	*6.9*	*11.0*	*10.8*	*2.3*
**4. Germ cell tumors**	18.6	7.5	2.4	11.0	6.6	8.6	7.6	7.7	0.8
**5. Soft-tissue Sarcomas**	5.3	10.0	7.3	4.2	4.6	9.3	6.9	7.1	0.5
**Total**	**117.1**	**166.6**	**107.4**	**106.9**	**129.6**	**117.7**	**123.8**	**126.3**	**1.1**

									
**State of México 96-02 (cases)**	**n = 44**	**n = 301**	**n = 234**	**n = 259**	**n = 446**	**n = 392**	**n = 838**	**n = 838**	**446/392**
**1. Leucemias**	23.7	84.1	52.8	52.6	63.6	54.9	59.3	60.2	1.2
**2. CNS tumors**	2.2	12.2	9.1	11.7	9.7	10.9	10.3	10.3	0.9
**3. Lymphomas**	0.0	6.1	15.0	10.9	13.8	6.7	10.3	9.9	2.1
*- Hodgkin lymphomas*	*0.0*	*1.5*	*7.1*	*6.5*	*5.9*	*3.9*	*4.9*	*4.6*	*1.6*
*- Non Hodgkin lymphomas*	*0.0*	*4.6*	*7.9*	*4.4*	*7.9*	*2.8*	*5.4*	*5.3*	*2.9*
**4. Germ cell tumors**	21.6	12.7	4.3	9.1	8.9	9.5	9.2	9.6	1.0
**5. Bone Tumors**	0.0	1.5	3.2	15.7	4.3	8.7	6.5	6.1	0.5
**Total**	**94.9**	**153.4**	**92.2**	**112.7**	**120.7**	**109.8**	**115.4**	**117.3**	**1.1**

									
**Morelos 96-02 (cases)**	**n = 9**	**n = 45**	**n = 32**	**n = 31**	**n = 60**	**n = 57**	**n = 117**	**n = 117**	**60/57**
**1. Leucemias**	0.0	82.2	73.9	60.2	64.2	72.5	68.3	66.8	0.9
**2. Lymphomas**	0.0	27.4	14.1	20.1	32.1	5.2	19.0	18.9	6.5
*- Hodgkin lymphomas*	*0.0*	*4.6*	*3.5*	*4.0*	*7.4*	*0.0*	*3.8*	*3.7*	*-*
*- Non Hodgkin lymphomas*	*0.0*	*22.8*	*10.6*	*16.0*	*24.7*	*5.2*	*15.2*	*15.1*	*5.0*
**3. CNS tumors**	52.2	22.8	7.0	8.0	9.9	18.1	13.9	15.7	0.6
**4. Renal Tumors**	52.0	18.3	3.5	0.0	9.9	7.8	8.9	10.8	1.3
**5. Bone Tumors**	0.0	4.6	7.0	16.0	4.9	12.9	8.9	8.3	0.4
**Total**	**235.1**	**205.5**	**112.6**	**124.4**	**148.2**	**147.7**	**148.0**	**154.3**	**1.1**

									
**Puebla 98-00 (cases)**	**n = 11**	**n = 29**	**n = 34**	**n = 40**	**n = 62**	**n = 52**	**n = 114**	**n = 114**	**62/52**
**1. Leukemias**	82.0	86.8	60.4	69.4	55.4	88.4	71.6	72.9	0.6
**2. Lymphomas**	0.0	9.1	24.9	40.8	37.8	10.4	24.3	22.7	3.8
*- Hodgkin lymphomas*	*0.0*	*4.6*	*14.2*	*20.4*	*20.1*	*5.2*	*12.8*	*11.9*	*4.0*
*- Non Hodgkin lymphomas*	*0.0*	*4.6*	*10.7*	*20.4*	*17.6*	*5.2*	*11.5*	*10.8*	*3.5*
**3. CNS tumors**	0.0	9.1	17.8	12.3	12.6	13.0	12.8	12.1	0.5
**4. Hepatic Tumors**	109.3	9.1	0.0	0.0	12.6	2.6	7.7	11.3	0.2
**5. Germ cell tumors**	27.3	4.6	0.0	16.3	2.5	13.0	7.7	8.3	0.2
**Total**	**300.7**	**132.4**	**120.7**	**163.4**	**156.0**	**135.2**	**145.8**	**150.7**	**1.2**

^1^Rates per 1,000,000 child/years; ^2^M/F = Ratio male/female; AAI = Average annual incidence; AAS = Average annual incidence standardized; CNS = Central Nervous System.

**Table 6:** Average annual and age-standardized incidence per period^1 ^of five principal malignant neoplasms in children served by IMSS and residing in the jurisdictions of Guerrero, Veracruz, Chiapas, and Yucatán

	**Age group (years)**				**Sex**				
**Diagnostic group**	**<1**	**1–4**	**5–9**	**10–14**	**M**	**F**	**AAI**	**AAS**	**M/F**^2^
**Guerrero 96-02 (cases)**	**N = 7**	**n = 51**	**N = 29**	**n = 38**	**n = 70**	**n = 55**	**n = 125**	**n = 125**	**70/55**
**1. Leukemias**	26.1	82.3	31.9	72.2	66.0	48.7	57.4	58.8	1.4
**2. CNS tumors**	0.0	38.8	28.3	31.6	15.8	46.0	30.7	30.3	0.4
**3. Lymphomas**	0.0	48.4	17.7	27.1	44.9	10.8	28.0	28.6	4.3
*- Hodgkin lymphomas*	*0.0*	*0.0*	*10.6*	*18.0*	*10.6*	*8.1*	*9.3*	*8.6*	*1.3*
*- Non Hodgkin lymphomas*	*0.0*	*48.4*	*7.1*	*9.0*	*34.3*	*2.7*	*18.7*	*19.9*	*0.9*
**4. Bone Tumors**	0.0	0.0	14.2	18.0	18.5	2.7	10.7	9.8	6.9
**5. Soft-tissue sarcomas**	52.1	0.0	0.0	18.0	10.6	5.4	8.0	9.3	2.0
**Total**	**182.5**	**247.0**	**102.7**	**171.4**	**184.7**	**148.7**	**166.9**	**173.5**	**1.3**

									
**Veracruz 98-02 (cases)**	**n = 2**	**n = 46**	**N = 44**	**n = 33**	**n = 66**	**n = 59**	**n = 125**	**n = 125**	**66/59**
**1. Leukemias**	40.9	67.3	57.4	42.2	53.0	55.1	54.0	54.8	1.0
**2. CNS tumors**	0.0	21.3	17.5	15.8	17.7	16.5	17.1	16.8	1.1
**3. Lymphomas**	0.0	3.5	22.4	15.8	21.2	7.3	14.4	12.9	3.0
*- Hodgkin lymphomas*	*0.0*	*3.5*	*10.0*	*10.6*	*12.4*	*3.7*	*8.1*	*7.4*	*3.5*
*- Non Hodgkin lymphomas*	*0.0*	*0.0*	*12.5*	*5.3*	*8.8*	*3.7*	*6.3*	*5.6*	*2.5*
**4. Germ cell tumors**	0.0	10.6	10.0	5.3	5.3	11.0	8.1	8.0	0.5
**5. Retinoblastoma**	0.0	21.3	0.0	0.0	3.5	7.3	5.4	6.6	0.5
**Total**	**40.9**	**163.0**	**109.7**	**87.1**	**116.5**	**108.3**	**112.5**	**114.3**	**1.1**

									
**Chiapas 96-02 (cases)**	**n = 12**	**n = 49**	**N = 39**	**n = 43**	**n = 73**	**n = 70**	**n = 143**	**n = 143**	**73/70**
**1. Leukemias**	55.1	96.6	62.4	68.5	69.2	76.2	72.7	74.2	0.9
**2. CNS tumors**	55.1	59.0	11.0	20.1	24.0	32.6	28.3	31.9	0.7
**3. Retinoblastoma**	137.8	32.2	3.7	0.0	13.3	19.0	16.1	21.8	0.7
**4. Lymphomas**	0.0	10.7	29.4	20.1	29.3	10.9	20.2	18.6	2.7
*- Hodgkin lymphomas*	*0.0*	*10.7*	*7.3*	*8.1*	*10.7*	*5.4*	*8.1*	*8.0*	*2.0*
*- Non Hodgkin lymphomas*	*0.0*	*0.0*	*22.0*	*12.1*	*18.6*	*5.4*	*12.1*	*10.6*	*3.4*
**5. Soft-tissue sarcomas**	0.0	37.6	0.0	8.1	8.0	16.3	12.1	14.0	0.5
**Total**	**330.7**	**263.0**	**143.2**	**173.2**	**194.4**	**190.4**	**192.4**	**203.5**	**1.0**

									
**Yucatán 98-00 (cases)**	**n = 3**	**n = 24**	**N = 15**	**n = 9**	**n = 24**	**n = 27**	**n = 51**	**n = 51**	**24/27**
**1. Leukemias**	72.7	73.8	49.5	19.4	42.1	55.6	48.8	50.1	0.8
**2. Germ cell tumors**	36.4	13.4	5.5	6.5	11.5	7.9	9.8	10.6	1.5
**3. CNS tumors**	0.0	13.4	16.5	0.0	11.5	7.9	9.8	9.5	1.5
**4. Bone Tumors**	0.0	0.0	0.0	25.9	0.0	15.9	7.8	7.5	-
**5. Neuroblastoma**	0.0	20.1	0.0	0.0	0.0	11.9	5.9	6.2	-
**Total**	**109.1**	**161.0**	**82.5**	**58.2**	**91.9**	**107.3**	**99.5**	**101.8**	**0.9**

^1^Rates per 1,000,000 child/years; ^2^M/F = Ratio male/female; AAI = Average annual incidence; AAS = Average annual age-standardized incidence; CNS = Central Nervous System.
